# Intranasal delivery of NGF rescues hearing impairment in aged SAMP8 mice

**DOI:** 10.1038/s41419-023-06100-8

**Published:** 2023-09-13

**Authors:** Vanessa Castelli, Michele d’Angelo, Francesca Zazzeroni, Davide Vecchiotti, Edoardo Alesse, Daria Capece, Laura Brandolini, Franca Cattani, Andrea Aramini, Marcello Allegretti, Annamaria Cimini

**Affiliations:** 1 Department of Life, Health and Environmental Sciences, University of L’Aquila, L’Aquila, Italy; 2 Department of Biotechnological and Applied Clinical Sciences, University of L’Aquila, L’Aquila, Italy; 3 Dompé Farmaceutici Spa, Via Campo di Pile 1, L’Aquila, Italy; 4 Sbarro Institute for Cancer Research and Molecular Medicine, Department of Biology, Temple University, Philadelphia, PA USA

**Keywords:** Neural ageing, Neurological disorders

## Abstract

Hearing loss impacts the quality of life and affects communication resulting in social isolation and reduced well-being. Despite its impact on society and economy, no therapies for age-related hearing loss are available so far. Loss of mechanosensory hair cells of the cochlea is a common event of hearing loss in humans. Studies performed in birds demonstrating that they can be replaced following the proliferation and transdifferentiation of supporting cells, strongly pointed out on HCs regeneration as the main focus of research aimed at hearing regeneration. Neurotrophins are growth factors involved in neuronal survival, development, differentiation, and plasticity. NGF has been involved in the interplay between auditory receptors and efferent innervation in the cochlea during development. During embryo development, both NGF and its receptors are highly expressed in the inner ears. It has been reported that NGF is implicated in the differentiation of auditory gangliar and hair cells. Thus, it has been proposed that NGF administration can decrease neuronal damage and prevent hearing loss. The main obstacle to the development of hearing impairment therapy is that efficient means of delivery for selected drugs to the cochlea are missing. Herein, in this study NGF was administered by the intranasal route. The first part of the study was focused on a biodistribution study, which showed the effective delivery in the cochlea; while the second part was focused on analyzing the potential therapeutic effect of NGF in senescence-accelerated prone strain 8 mice. Interestingly, intranasal administration of NGF resulted protective in counteracting hearing impairment in SAMP8 mice, ameliorating hearing performances (analyzed by auditory brainstem responses and distortion product otoacoustic emission) and hair cells morphology (analyzed by microscopy analysis). The results obtained were encouraging indicating that the neurotrophin NGF was efficiently delivered to the inner ear and that it was effective in counteracting hearing loss.

## Introduction

Hearing loss is a common sensory disorder characterized by sensorineural hearing loss generally starting in individuals over 65 years. It impacts the quality of life and affects communication resulting in social isolation and reduced well-being [1, 2]. The major site of the auditory system that is affected by aging is the cochlea, where the loss of hair cells (HCs) and spiral ganglion neurons (SGNs) occurs. Aging affects all the auditory system, also the peripheral and central auditory systems. In spite of its impact on society and economy [3], no therapies to prevent or counteract age-related hearing loss (ARHL) are available.

In the past two decades, an important piece of studies has been devoted to hearing research and to the comprehension of biological mechanisms contributing to hearing loss [4]. Hearing loss can result from several types of insults affecting the different cochlear cell types. In the ARHL, injury to the HCs (in particular outer hair cells (OHCs)) has been the issue; new findings point toward the neurodegeneration of the nervous component of the cochlea as the crucial factor in the disease.

While the cellular and molecular mechanisms of ARHL are poorly understood, preclinical and clinical studies reported that loss of synapses between inner hair cells (IHCs) and SGNs, also known as cochlear or IHC synaptopathy, is an early pathology that precedes IHC loss in mice [5] and humans [6–8].

Loss of mechanosensory HCs of the cochlea is a common event of hearing loss in humans [9]. Studies performed in the birds demonstrating that they can be replaced following the proliferation and transdifferentiation of supporting cells, strongly pointed out on HCs regeneration as the focus of research aimed at hearing regeneration. Neurotrophic factors control the survival and differentiation of neurons during neurodevelopment and are also crucial for maintaining the synaptic connectivity and plasticity in the adult nervous system [10]. Four members have been described: nerve growth factor (NGF), brain-derived neurotrophic factor (BDNF), neurotrophin-3 (NT-3), and neurotrophin-4/5 (NT4/5). In particular, NGF has been involved in the interplay between auditory receptors and efferent innervation in the cochlea during development [11]. Several studies have reported that NGF signaling through Tropomyosin receptor kinase (TrkA) receptors phosphorylation activates the downstream Akt activation determining neuronal survival [12].

During embryo development, both NGF and its receptors are highly expressed in the inner ears. It has been reported that NGF is implicated in the differentiation of auditory gangliar and HCs [13]. Thus, it has been proposed that NGF administration can decrease neuronal damage and prevent hearing loss [14]. Mouse NGF (mNGF) ameliorative effects on auditory dysfunction in DBD/2J mice, a progressive hearing loss model, has been previously published and proposing possible mechanism(s) of mNGF in the protection of auditory functions [14], indicating decrease of Bak/Bax and Caspase activation, resulting in the suppression of the apoptotic pathway.

In this work, the effect of NGF has been studied in an animal model of ARHL, using the senescence-accelerated prone strain 8 (SAMP8) mice. SAMP8 mice are a strain derived from AKR/J mice, chosen for a phenotype toward either accelerated senescence with a mean life span of 9 months [15]. It has been shown that the SAMP8 strain presents progressive degeneration of OHCs, SGNs, stria vascularis, and finally IHCs that resembles human ARHL [16].

The main obstacle to the development of hearing impairment therapy is that efficient means of delivery for selected drugs to the cochlea [17] are missing. Like the blood–brain barrier in the central nervous system (CNS), the blood–labyrinth barrier (BLB) defends the cochlea and vestibular system resulting in limited drug exposure in the inner ear after systemic administration [18]. To test the potential therapeutic effects of neurotrophins, increasing studies have focused on the exogenous delivery (by osmotic pump or micro cannulation) in numerous animal models, and interestingly, they resulted effective in preserving the auditory nerve function from both drug-induced ototoxicity and noise exposure-induced hearing loss and can increase cochlear neuron survival [19–21]. Other interesting studies showed that local administration of exogenous NGF stimulated extensive neurite projections of dorsal root ganglion once transplanted into adult rat cochlea [22–25].

Therefore, the local route to the inner ear may overcome the toxic effects due to the systemic drug administration allowing drug repurposing for hearing loss. The possibility of a minimally invasive alternative route of administration may have the potential to transform not only the therapeutic approaches of hearing impairment but also the related unmet health demands of tinnitus and balance disturbs as that intravitreal administration has revolutionized the treatment of retinal disorders. To address this issue, herein, NGF was administered by the intranasal route, and in the first part of the study a biodistribution study was performed in C57BL/6, showing the effective delivery in the cochlea. In the second part of the study, the therapeutic potential of intranasal administration of NGF was evaluated. Notably, the neurotrophin was able to counteract ARHL, ameliorating hearing performances and morphological changes. The results obtained were encouraging indicating that the neurotrophin NGF was efficiently delivered to the inner ear and thus proposing NGF as an efficient effector for the protection and recovery of hearing loss.

## Materials and methods

### Biodistribution study

For this study as strain, C57BL/6 males were selected (*n* = 19). Mice were housed in macrolon cages with filter hoods, in a room where the air was continuously filtered, thereby avoiding contamination. All experiments were performed in the compliance with the European Directives (Reference Number: D3417223, APAFIS#23920- 2020020320279696 v3). Animals arrived on site 4 days before compound administration to allow optimal acclimation. During experiments, paired animals were caged at a controlled temperature with a day/night cycle of 12/12 h. Animals received water (control tap water) and nutrition ad libitum. Animal were purchased from Envigo, USA. Animals were randomly distributed over the different experimental conditions and blinding was applied during data acquisition and analysis when possible.

**Fig. 1:**
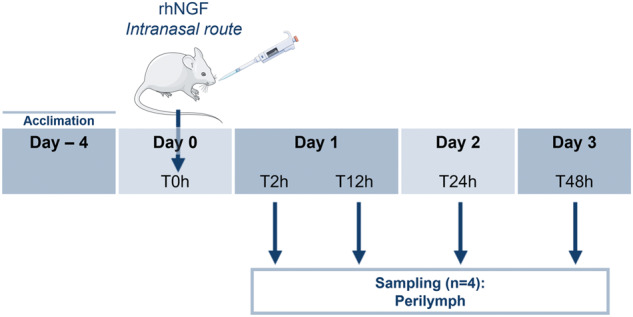
Biodistribution study scheme. Schematic representation of the biodistribution of NGF in mice upon intranasal administration at different time points.

Mice were 10 weeks old and were divided into 5 groups, (1) vehicle that received only saline buffer and sampling at 12 h (*n* = 3), (2) NGF treated and sampling at 2 h (*n* = 4), (3) NGF-treated and sampling at 12 h (*n* = 4), (4) NGF compound treated and sampling at 24 h (*n* = 4), (5) NGF compound treated and sampling at 48 h (*n* = 4) (Fig. 1). In all, 10 µL NGF at the concentration of 2 mg/mL was administered by the intranasal route. Human recombinant NGF was provided by Dompé Farmaceutici Spa.

### Intranasal compound administration

Using a dominant hand, the micropipette was led with 10 μL of compound or vehicle. The tip of the pipette was positioned near the mouse’s left nostril at a 45-degree angle. The droplet was put near enough to the mouse’s nostril in order to facilitate the inhalation of the droplet. Immediately after the mouse inhaled this small drop, and about 2–3 s later another drop was dispensed. After administration, the mouse was maintained in this position for 15 s.

### Perilymph sampling

Each mouse was sacrificed by decapitation and the tympanic bullas were sampled. Each tympanic bulla was opened, and the cochlea was cleaned. Then, using a capillary micropipette of 5 μL, the perilymph was sampled from the base. Left and right cochleae were used as duplicates. Around 1 µL of perilymph was sampled per cochlea per animal and stored at −20 °C for hNGF ELISA analysis.

### Human NGF analysis by ELISA

In all, 10 µL of the perilymph diluted samples at 1/10 were analyzed by ELISA method (Novus Biological, Ref. NBP2-62776). Standard references were diluted at 1000, 500, 250, 125, 62.5, 31.25, 15.63, and 0 pg/mL to perform the standard curve. The Standard working solution at various concentrations was added into two wells and the samples were to other wells. The plate was sealed and incubated for 1 h and 30 min at 37 °C. Then the liquid of each well was aspirated, and immediately 100 μL of Biotinylated Detection solution was put into each well. The plate was again sealed and incubated for 1 h at 37 °C. After incubation, the solution from each well was removed and 350 μL of wash buffer was added. After extensive washes, 100 μL of HRP-conjugated working solution was added to each well. The plate was again sealed and incubated for 30 min at 37 °C. After washes, 90 μL of Substrate Reagent was put in each well and incubated for 15 min at 37 °C. Finally, 50 μL of Stop Solution was added to each well and read at microplate reader at 450 nm.

### Aged animals

**Fig. 2:**
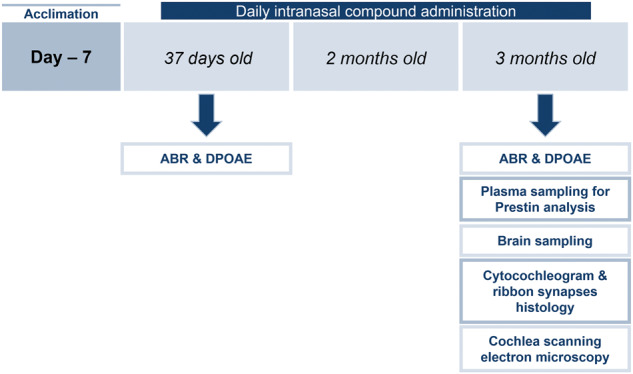
Aged animal study and NGF treatment scheme. NGF was administered intranasally in SAMP8 mice and then different tests were performed at different time points.

SAMP8 and SAMR1 (their senescence resistant control mice (SAMR1) male 1-month-old were purchased from Envigo (*n* = 24). All experiments were performed in the compliance with the European Directives (Reference Number: D3417223, APAFIS#23920- 2020020320279696 v3). Mice were housed in macrolon cages with filter hoods, in a room where the air is continuously filtered, thereby avoiding contamination. Animals were randomly distributed over the different experimental conditions and blinding was applied during data acquisition and analysis when possible. Mice were divided in Group 1: SAMR1 (negative control *n* = 8), Group 2: SAMP8 + vehicle (*n* = 8) and, Group 3: SAMP8 + NGF (*n* = 8). NGF was daily administered by intranasal route (10 µL) (Fig. 2).

### Auditory brainstem responses (ABRs)

ABRs are electric potentials recorded from scalp electrodes, and the first ABR wave correspond to the total activity of the auditory nerve fibers communicating the IHCs. For the procedure, animals were anesthetized using a ketamine/xylazine mixture, and body temperature was maintained at 37 °C using a heating pad. Earphones were positioned in the left ear of each mouse, an active electrode was positioned in the vertex of the skull, a reference electrode under the skin of the mastoid bone, and a ground electrode in the neck skin. The stimuli involved tone pips of five frequencies (2–24 kHz) at various sound levels (from 0 to 90 dB) varying to cover the mouse auditory frequency range. ABR analysis of each mouse were executed individually and using the OtoPhyLab system. Evoked potentials were obtained by the signal averaging technique for each noise level and ABR thresholds for each frequency were revealed using OtoPhyLab software.

### Distortion product otoacoustic emission (DPOAE)

DPOAEs are acoustic signals produced and amplified by the cochlear epithelium, suggesting an index of cochlear functionality. They are linked to OHCs health which amplifies sound-evoked cochlear vibrations. They are independent from IHCs or auditory nerve fibers. For the procedure, animals were anesthetized using a ketamine/xylazine mixture, and a probe (OtoPhyLab) was introduced into the external left ear canal. The primary tone F2 was set at five frequencies (4–kHz) at 58 dB. The frequency ratio F2/F1 was set at 1.2. At all frequencies (F2), the input of DPOAE systems was received, digitized, and calculated using the output of the microphone. The amplitudes of the frequency component at the distortion product frequency were established and represented.

### Prestin quantification

For each animal, 2 mL of blood was collected by cardiac puncture and put in a tube containing EDTA and immediately centrifuged for 15 min at 1000 × *g* at 2–8 °C. The supernatant (plasma) was kept at −80 °C until analysis. Prestin quantification for each animal was performed by the ELISA method (Mybiosource, MBS286559).

### Cytocochleogram and ribbon synapses

Left cochleae of all mice were extracted and fixed with paraformaldehyde solution overnight and then, decalcified for 7 days in EDTA. The membranous and sensory spiral enclosing the organ of Corti were isolated and the HCs were immunolabeled for anti-Myosin-VIIa (Abcam Catalog #ab150386). The medial region of the OC was mounted on glass slides and observed using a confocal microscope. The total number of IHCs and OHCs was counted for the mid-segment. For the ribbon synapse immunostaining, the right cochleae of 1 animal/group were sampled, fixed, and decalcified as previously stated, and the ribbon synapses of the mid-segment were immunolabeled for anti-GluR2 (Invitrogen Catalog # 32-0300) and the cochlear neurons using anti-Tuj1 (Invitrogen Catalog # 32-0300). Samples were mounted on glass slides and observed using a confocal microscope. Processing and analysis of immunohistochemistry images were performed using ImageJ with Fiji. The number of ribbon synapses immunostained for GluR2 and was counted using the Analyze Particles Plugin.

### Cochlea scanning electron microscopy imaging

Right cochleae of 3 animals/group were extracted from the temporal bone and fixed in 2.5% glutaraldehyde in PHEM buffer (PIPES 60 mM, HEPES free acid 25 mM, EGTA 20 mM, MgCl_2_ 2 mM) overnight at RT. The stria vascularis, tectorial, and Reissner’s membranes were separated by microdissection. After washing in PHEM buffer, samples were dehydrated in a graded series of ethanol (30–100%), critical point dried in CO_2_, coated with gold palladium, and examined using a scanning electron microscope Hitachi S4000.

### Statistical evaluation

Descriptive statistics by groups were expressed as mean ± SEM for continuous variables. Statistical significances were calculated using two-way or one-way ANOVA, and by a Turkey’s multiple comparisons post hoc test. Statistical analyses were performed using GraphPad Prism (GraphPad Software, La Jolla, CA, USA). A *P* value of <0.05 was set as significant.

## Results

### Body weight and clinical signs

No mortality and pathological clinical signs were observed between groups at the analyzed time points. As expected, a significant reduction in body weight was observed in the SAMP8+vehicle group compared to the SAMR1 control group from day 33 (Supplementary Fig. 1). Interestingly, a substantial rise in body weight was observed in the SAMP8+rhNGF-treated group in comparison with the SAMP8+vehicle group suggesting a direct or indirect effect of the compound administration on animal body weight loss induced by aging (Supplementary Fig. 1).

### Human NGF in perilymph

**Fig. 3:**
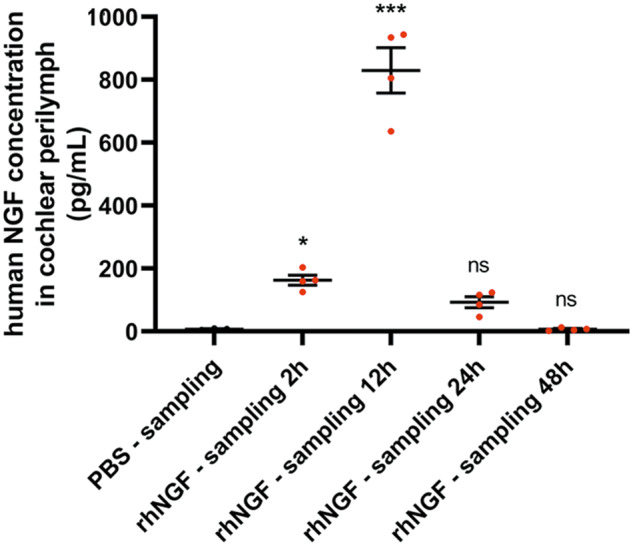
Graphical representation of the human NGF concentration in cochlear perilymph at 12, 24, and 48 h post-treatment in rhNGF- treated (red) and PBS-treated (black) animals. Data are mean ± SEM. *<0.05; ***<0.0001 vs PBS-sampling 12 h. *N* = 4.

A significant increase in human NGF concentration was observed in perilymph starting at 2 h post-treatment in the NGF-treated group compared to the vehicle-treated group. 2 h after single NGF administration, the compound was detected in perilymph at a mean of 162.7 pg/mL, 12 h the compound was detected in perilymph at a mean of 832.2 pg/mL. A progressive decrease in NGF concentration was observed at 24 and 48 h post-treatment with a concentration of 92.8 and 6.8 pg/mL, respectively (Fig. 3). Even if a slight increase in NGF concentration was observed at 24 h post-treatment, this concentration was not statistically different compared to the vehicle-treated group. Taken together, these results demonstrated that NGF can reach the cochlear perilymph when administrated by the intranasal route with a peak concentration at 12 h post-treatment at the experimental conditions.

### NGF reduces age-related ABR threshold shifts in SAMP8 mice

**Fig. 4:**
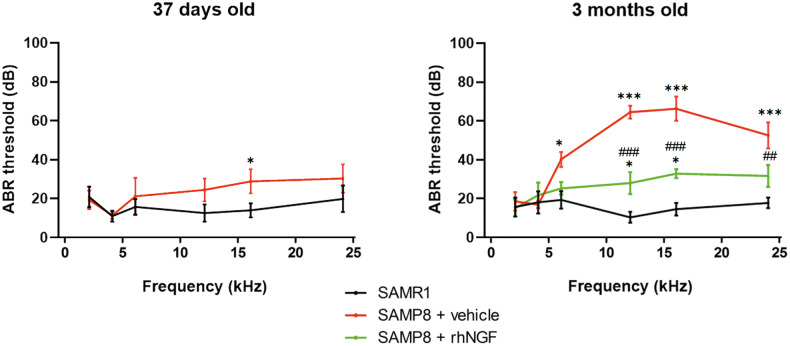
Graphical representation of ABR threshold at 1 and 3 months old. Data are mean ± SEM. *N* = 8.Graphical representation of ABR threshold at 1 and 3 months old for the SAMR1 (left), SAMP8+vehicle (mid), and SAMP8+rhNGF (right) groups. Data are mean ± SEM. *<0.05; ***<0.0001 vs SAMR1. ^##^<0.005; ^###^<0.0001 vs SAMP8 + vehicle. *N* = 8.

ABR thresholds were analyzed, and a significant difference was detected at 37 days old SAMP8 mice, indicating an impairment of hearing functions. As expected, a substantial rise in ABR thresholds was observed for the SAMP8+vehicle group compared to the SAMR1 control group at 6, 12, 16, and 24 kHz at 3 months old (Fig. 4). The SAMP8 animals treated with NGF showed a significant decrease of ABR threshold at 12, 16, and 24 kHz compared to the SAMP8+vehicle group (Fig. 4) suggesting a protective effect of the compound on the ARHL. Moreover, statistical differences in ABR threshold were observed between SAMP8 + NGF and SAMR1 groups at 12 and 16 kHz, suggesting a partial efficacy of the compound on ARHL at these experimental conditions.

### NGF increases DPOAE

**Fig. 5:**
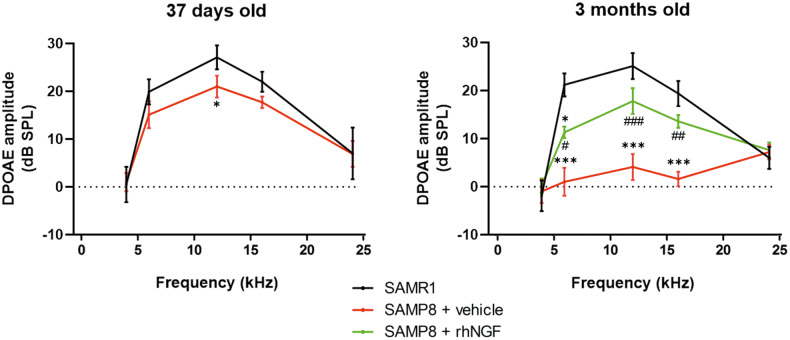
Graphical representation of DPOAE amplitudes at 1 and 3 months old. Data are mean ± SEM. *N* = 8. Graphical representation of DPOAE amplitudes at 1 and 3 months old for the SAMR1 (left), SAMP8+vehicle (mid), and SAMP8+rhNGF (right) groups. Data are mean ± SEM. *<0.05; ***<0.0001 vs SAMR1. ^#^<0.05; ^###^<0.0001 vs SAMP8 + vehicle. ***<0.0001 vs SAMR1. ^###^<0.0001 vs SAMP8 + vehicle. *N* = 8.

Similar DPOAE amplitudes were observed at the baseline for the three groups at the analyzed frequencies demonstrating the functional OHC integrity at this time point. The functional OHC integrity was significantly affected in 37 days old SAMP8 animals as highlighted by altered DPOAE amplitude. Moreover, a strong reduction in DPOAE amplitude was observed for the SAMP8+vehicle group compared to the SAMR1 control group at 6, 12, and 16 kHz in 3 months mice (Fig. 5). The SAMP8 animals treated with NGF presented a significantly higher DPOAE amplitude at 6, 12, and 16 kHz compared to the SAMP8+vehicle group (Fig. 5), confirming a direct or indirect protective role of the compound on OHC functionality leading to hearing improvement in aged animals. Compared to the SAMR1 control group, the SAMP8 + NGF group presented a slight but no significant decrease of the DPOAE amplitude at the analyzed frequencies except at 6 kHz suggesting a partial efficacy of the compound in these experimental conditions.

### NGF reduces plasma prestin levels

**Fig. 6:**
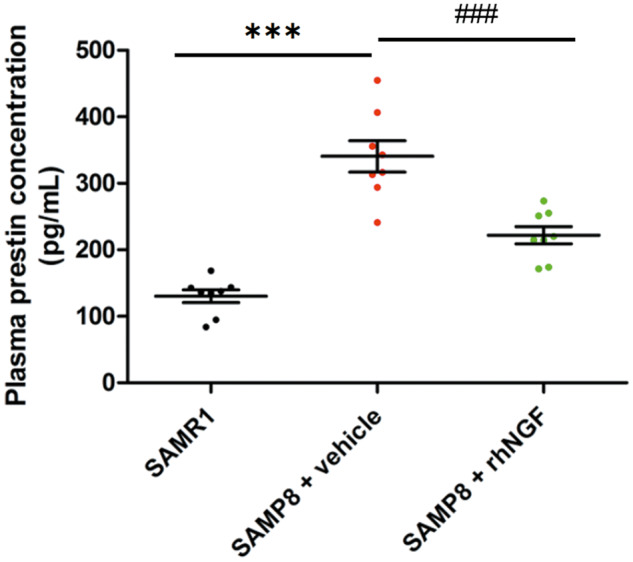
Graphical representation of plasma prestin concentration. Data are mean ± SEM. ***<0.0001 vs SAMR1. ^###^<0.0001 vs SAMP8 + vehicle. *N* = 8.

Prestin is a recognized biomarker for cochlear damage [26]. A significant increase of plasma prestin concentration was observed in the SAMP8+vehicle group compared to the SAMR1 at 3 months old (Fig. 6) suggesting cochlear damage and corroborating electrophysiological and otoacoustic readouts. The SAMP8 animals treated with NGF presented significantly lower plasma prestin concentration compared to the SAMP8+vehicle group, even if the concentration of the NGF-treated group remains statistically higher than the SAMR1 control group (Fig. 6). Taken together, these data agree with the protective role of the compound on hearing impairment induced by aging.

### NGF reduces HC loss

**Fig. 7:**
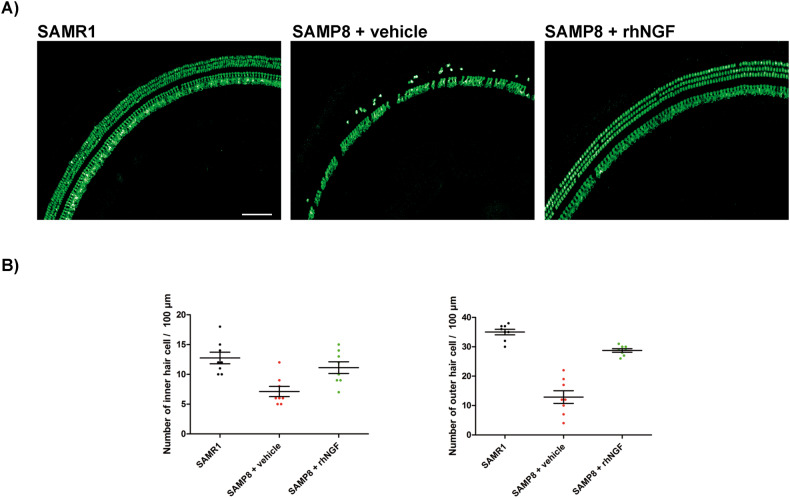
Cytocochleogram analysis and relative graphical representations. **A** Representative images for cochlear segments (scale bar: 100 µm) stained with Myosin VIIa of SAMP8 mice control, vehicle and hrNGF-treated are shown. **B** Graphical representation of the number of IHC (left) and OHC (right) at 3 months. *N* = 8. Bar = 100 μm.

To study the effect of NGF on HC survival, cochleae were isolated for immunohistochemical evaluation after the final follow-up ABR measurement. Cytocochleograms were produced from OC whole-mount preparations marked with Myosin VIIa. Representative images are shown in Fig. 7A. Quantification of the total number of IHCs and OHCs was performed by the immunohistology analysis of the mid-turn of each cochlea. A significant reduction in the total number of OHC and IHC was detected in the SAMP8+vehicle group compared to the SAMR1at 3 months old, confirming the cochlear damage induced by senescence. The SAMP8 animals treated with NGF presented a significant increase in the number of IHCs and OHCs compared to the SAMP8+vehicle group (Fig. 7B). Even if the number of OHCs of the SAMP8 + NGF group was statistically lower than the SAMR1 group, no significant differences in the total number of IHCs were observed at this time point. Taken together, these results confirm the positive efficacy of the compound on ARHL when administrate by intranasal route once a day for 2 months.

**Fig. 8:**
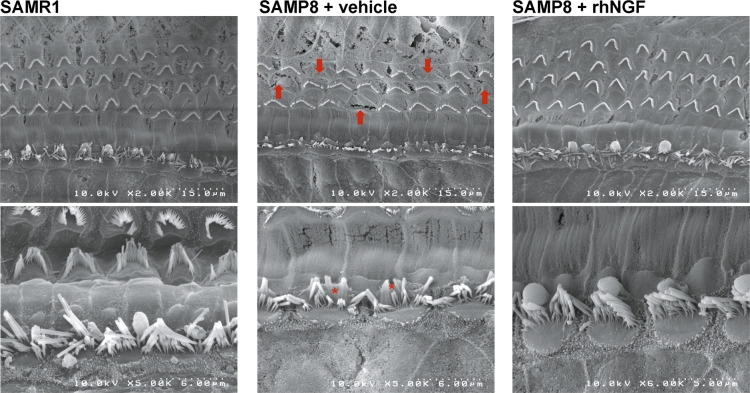
SEM images of the cochlea mid-turn of SAMR1 SAMP8+vehicle and SAMP8+rhNGF groups. Arrows indicate hair cell loss. Asterisks indicate a fusion of stereocilia.

OHC and IHC stereocilia morphology was determined by scanning electron microscopy (SEM) in the mid-turn of the cochlea. OHCs loss was confirmed in the SAMP8 animals treated with vehicle (arrows in Fig. 8), whereas no cell loss was observed in SAMR1 control animals or SAMP8 animals treated with NGF. In line with the literature [16], IHCs stereocilia fusion was observed in the SAMP8 animals treated with vehicle (asterisk in Fig. 8), whereas normal stereocilia morphology was observed in the SAMP8 animals treated with NGF. The morphology of stereocilia of the SAMP8 + NGF group was close to SAMR1 control animal morphology confirming the protective efficacy of the compound in these experimental conditions. Some blood cells probably infiltrated into the cochlea during animal perfusion or dissection were observed in all images.

**Fig. 9:**
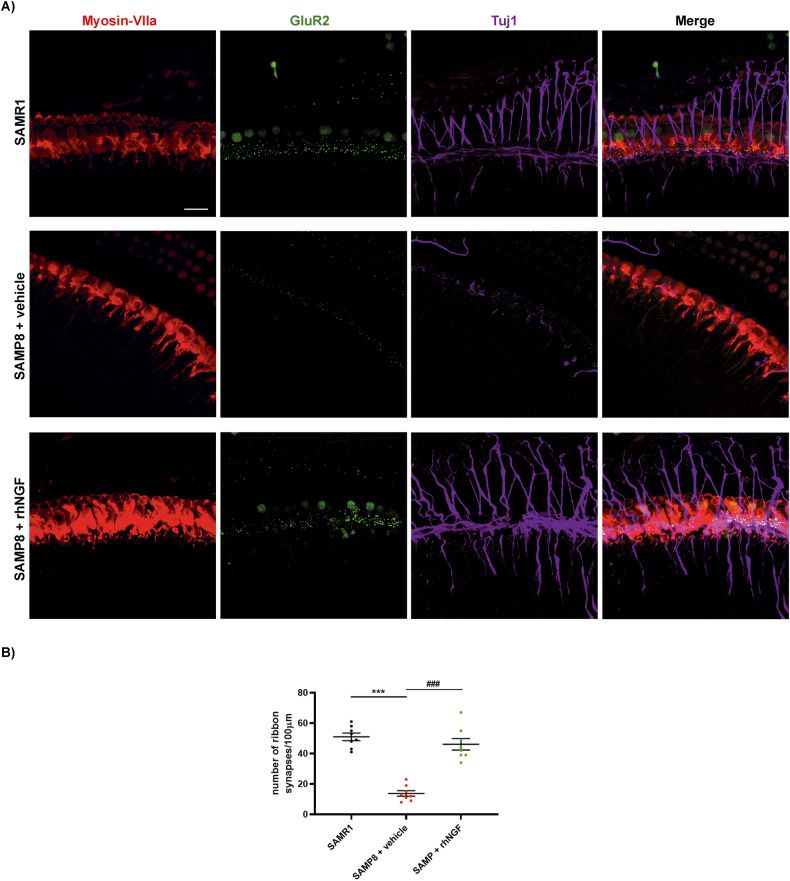
Immunofluorescence analysis in mice cochlea. **A** Immunofluorescence images of cochlea mid-turn of SAMR1, SAMP8+vehicle, and SAMP8+rhNGF groups at 3 months old. Myosin-VIIa marks hair cells (magenta), GluR2 marks ribbon synapses (green), and Tuj1 marks efferent and afferent neurons of the cochlea (blue). Merge compositions on the right. Bar = 35 μm. **B** The number of ribbon synapses of the IHCs per 100 µm of sensory epithelium. Data are mean ± SEM. ***<0.0001 vs SAMR1. ^###^<0.0001 vs SAMP8 + vehicle. *N*  = 8.

To investigate the potential role of synaptopathy prevention by NGF, Glur2 receptor was immunolabeled, together with MyosinVII, a typical marker of IHC and Tuj1, a marker of neurons were used. Imaging of ribbon synapses and cochlear neurons was performed by immunolabeling the cochlear mid-turn. As expected, a strong reduction in the number of ribbons synapses, and neurons was observed in the cochlea of SAMP8 mouse treated with the vehicle at 3 months old (Fig. 9). Notably, postsynaptic GluR2 puncta was significantly greater in the NGF treated group compared to the control group (Fig. 9B). The morphology of the SAMP8 + NGF mouse was similar to the SAMR1 control animal confirming the protective efficacy of the compound in preserving the morphology in these experimental conditions.

## Discussion

Hearing impairment is a relevant health problem due to numerous exogenous insults that induce inflammation and oxidative stress, leading to cell injury and hearing loss. Aging, radiation exposure, acoustic trauma, and drug exposure can trigger ROS generation in the inner ear with loss of sensory cells and hearing impairment. Hearing impairment is the third most frequent sensory disorder in humans, with ARHL establishing the primary reason in the population older than 70 years [27]. Even if ARHL is an emerging health problem, there is no effective therapeutic approach to treat or counteract this disorder.

External sounds are converted by the tympanic membrane and the middle ear ossicles to the oval window to generate motion of perilymph and endolymph fluids in the inner ear fluids. Therefore, HCs in the OC reveal the fluid motion through stereocilia, mechanoelectrical transduction (MET) channels open, leading to the entry of potassium from the potassium-rich endolymph, depolarizing the HCs to stimulate the production of the neurotransmitter glutamate at their ribbon synapses [28]. The organ of Corti contains two types of HCs, OHCs, and IHCs arranged along the basilar membrane enabling the precise distinction of the range of audible frequencies. Both types of HCs are in communication with various efferent neural cells s that support feedback loops from the CNS to the cochlea. Numerous supporting cells are present that are involved in the maintenance of HC function, structure, and homeostasis. HC damage represents the main cause of audiometric threshold increase in sensorineural hearing impairment, and ototoxicity [29]. Studies performed in the birds demonstrating that the lost HCs can be regenerated via the proliferation and transdifferentiation of supporting cells [30–32], strongly pointed out on HCs regeneration as the main focus of research aimed at hearing regeneration.

The ribbon synapses connecting IHCs and SGN afferent fibers are susceptible to ototoxic agents, aging, and noise damage. This has been observed in in vivo models of noise-induced impairment in which a favored and irreversible loss of a subpopulation of IHC afferent synapses, those responsible for the so-called high threshold, low spontaneous activity fibers [33, 34] has been reported. Favored susceptibility of ribbon synapses has also been described in post-mortem studies in temporal bone samples, where during aging the loss of type I afferent fibers and IHC synapses significantly leads the loss of the IHCs [7, 8].

Injury to ribbon synapses was initially identified as “hidden hearing loss,” a damage of hearing quality appearing in presence of a normal audiometric hearing thresholds. emerging studies in the field suggests that this alteration in neuronal connectivity is at basis of auditory problems, distinguished by a failure to hear sounds such as a conversation, in the presence of background noise [33, 34].

Similar to the BBB, BLB defends the cochlea and vestibular system causing limiting the drug to reach the inner ear upon systemic administration [35]. This has prompted new research aims to identify new no-invasive administration routes, identifying local administration of drugs, i.e., intratympanic injection or local administration by the oval window, as new preferable administration routes.

Despite promising results obtained with drug intratympanic administration, it appears as an invasive method and leakage of liquid preparations through the Eustachian tube is the most important limit of this route. Thus, finding an alternative route of administration that effectively deliver the drug and is minimally invasive is challenging.

Indeed, the main strength of this study is that we selected the intranasal route of administration and that it was effective in delivering NGF in the target area (patent No. EP22188990.0 (05-08-2022).

This choice is derived from the analysis of previous reports demonstrating that the perilymph in the tympanic scale derives from cerebrospinal fluid (CSF) and possesses the same composition. Moreover, it is known that the perilymph in the scala vestibuli comes from blood plasma across a blood-perylimph barrier, whereas that of the scala tympani originates from CSF. In fact, the cerebrospinal fluid–perilymph barrier comprises the cochlear aqueduct [32], which joins CSF of the subarachnoid space to the perilymph of the scala tympani and offers the possibility of interconnection between the two fluids [36]. Thus, since in rabbit (data not shown) we found an enrichment of NGF in the CSF upon intranasal administration, it may be conceivable that the enrichment of NGF that we found in the cochlea may arrive from CSF. However, the whole auditory system from the ear to the auditory cortex are involved in sound perception. The ARHL affects the whole auditory system, both the peripheral and central auditory systems [37]. Other reports have indicated age-related changes in the superior olivary formation, inferior colliculus, and auditory cortex of the central auditory system [38–40]. Generally, ARHL results in atrophy of the auditory cortex accompanied by changes in connectivity between functional networks [41], including peripheral auditory structures, potentially compromising hearing processing [42]. It is conceivable that, since rhNGF, upon intranasal administration, appears distributed also in brain, the neurotrophic signal may arrive to the HCs also by afferent neuronal fibers.

Our results provide the first demonstration that NGF, reaching the cochlea, exerted significative protective effects in preserving HCs and number, as well as ribbon synapses and Glutamate receptors, thus indicating that the intranasal administration is a less invasive and more efficient administration route.

In this study, SAMP8 mice were selected as a model to detect the otoprotective effect of NGF and, notably, the neurotrophin could preserve the function of OHCs safeguarding hearing loss in aging mice. Specifically, electrophysiology studies reported a protective effect in animals treated with NGF both at the functional and morphological levels. In this condition, NGF efficiently decreased click-evoked ABR threshold shifts compared to vehicle-treated ears. Interestingly, SAMP8 mice treated with NGF once a day for two months by intranasal route presented a significant decrease in ABR threshold, an increase of DPOAE amplitude, and a decrease of plasma prestin and cochlear HC loss at 3 months old compared to the vehicle group, paralleled by the protection of HCs, particularly of OHCs, as highlighted by cytocochleogram. Moreover, the histological characterization by SEM and immunohistochemistry of cochleae demonstrated the absence of stereocilia fusion of IHCs and an increase of the number of ribbon synapses and cochlear neurons after the compound treatment corroborating electrophysiology, otoacoustic and functional data. These results are in agreement with previous observations on DBA/J2 mice treated with mouse NGF, where an antiapoptotic and pro-survival role has been proposed for this neurotrophin [14].

Our results provide for the first time, evidence that the intranasal administration route efficiently targets the cochlea promoting the maintenance of IHC, OHC, and ribbon synapses and their functionality during aging, supporting the potential of NGF administration as a strong therapeutic approach to reduce the burden of ARHL also in humans.

Taken together, these results confirm that the NGF performs significant protective efficacy on age-related hearing impairment by ameliorating hearing performances and preserving histopathology of the cochlea in the preclinical model of accelerated senescence SAMP8.

## Supplementary information

Supplementary figure

AJ checklist

## Data Availability

Data will be provided by corresponding authors upon requests.
