# Supplementary material for: Intranasal delivery of NGF rescues hearing impairment in aged SAMP8 mice
Source: Cell Death Dis. 2023 Sep 13;14(9):605. doi: 10.1038/s41419-023-06100-8 (PMC10499813; doi:10.1038/s41419-023-06100-8)
Supplement: Supplementary file 1 — Supplementary figure [file 41419_2023_6100_MOESM1_ESM.docx]

Supporting Information for

**Intranasal delivery of NGF rescues hearing impairment in aged SAMP8 mice**

Vanessa Castelli^1*^, Michele d’Angelo^1*^, Francesca Zazzeroni^2§^, Davide Vecchiotti^2^, Edoardo Alesse^2^, Daria Capece^2^, Laura Brandolini^3^, Franca Cattani^3^, Andrea Aramini^3^, Marcello Allegretti^3^, Annamaria Cimini^1,4§^,

^1^Dept of Life, Health and Environmental Sciences, University of L’Aquila

^2^Dept of Biotechnological and Applied Clinical Sciences, University of L’Aquila

^3^Dompé Farmaceutici Spa, Via Campo di Pile 1, L’Aquila

^4^Sbarro Institute for Cancer Research and Molecular Medicine, Dept of Biology, Temple University, Philadelphia, USA

* These authors contributed equally to this work

§Corresponding Authors: Annamaria Cimini, Dept of Life, Health and Environmental Sciences, University of L’Aquila; e-mail: [annamaria.cimini@univaq.it](mailto:annamaria.cimini@univaq.it); Francesca Zazzeroni, Dept of Biotechnological and Applied Clinical Sciences, University of L’Aquila; e-mail: [francesca.zazzeroni@univaq.it](mailto:francesca.zazzeroni@univaq.it)

This PDF file includes:

Figure S1


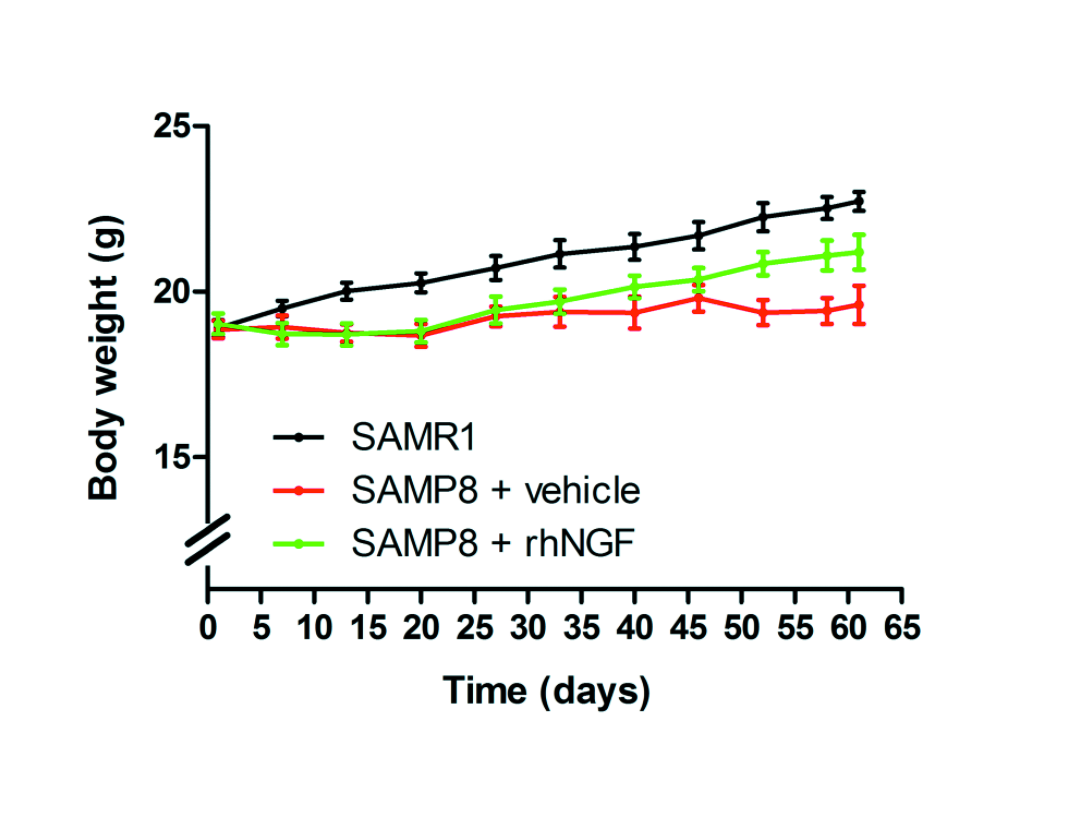


**Suppl fig 1**: Graphical representation of animal body weight. Data are mean±SEM. N=4
